# Development of a transformer model for predicting the prognosis of patients with hepatocellular carcinoma after radiofrequency ablation

**DOI:** 10.1007/s12072-023-10585-y

**Published:** 2023-09-09

**Authors:** Masaya Sato, Makoto Moriyama, Tsuyoshi Fukumoto, Tomoharu Yamada, Taijiro Wake, Ryo Nakagomi, Takuma Nakatsuka, Tatsuya Minami, Koji Uchino, Kenichiro Enooku, Hayato Nakagawa, Shuichiro Shiina, Kazuhiko Koike, Mitsuhiro Fujishiro, Ryosuke Tateishi

**Affiliations:** 1 Department of Clinical Laboratory Medicine, Graduate School of Medicine, The University of Tokyo, 7-3-1 Hongo, Bunkyo-Ku, Tokyo, 113-8655 Japan; 2 Department of Gastroenterology, Graduate School of Medicine, The University of Tokyo, Tokyo, Japan; 3 Department of Gastroenterology, Juntendo University, Tokyo, Japan

**Keywords:** Hepatocellular carcinoma, Radiofrequency ablation, Transformer, Prognosis

## Abstract

**Introduction:**

Radiofrequency ablation (RFA) is a widely accepted, minimally invasive treatment modality for patients with hepatocellular carcinoma (HCC). Accurate prognosis prediction is important to identify patients at high risk for cancer progression/recurrence after RFA. Recently, state-of-the-art transformer models showing improved performance over existing deep learning-based models have been developed in several fields. This study was aimed at developing and validating a transformer model to predict the overall survival in HCC patients with treated by RFA.

**Methods:**

We enrolled a total of 1778 treatment-naïve HCC patients treated by RFA as the first-line treatment. We developed a transformer-based machine learning model to predict the overall survival in the HCC patients treated by RFA and compared its predictive performance with that of a deep learning-based model. Model performance was evaluated by determining the Harrel’s c-index and validated externally by the split-sample method.

**Results:**

The Harrel’s *c*-index of the transformer-based model was 0.69, indicating its better discrimination performance than that of the deep learning model (Harrel’s *c*-index, 0.60) in the external validation cohort. The transformer model showed a high discriminative ability for stratifying the external validation cohort into two or three different risk groups (*p* < 0.001 for both risk groupings). The model also enabled output of a personalized cumulative recurrence prediction curve for each patient.

**Conclusions:**

We developed a novel transformer model for personalized prediction of the overall survival in HCC patients after RFA treatment. The current model may offer a personalized survival prediction schema for patients with HCC undergoing RFA treatment.

**Supplementary Information:**

The online version contains supplementary material available at 10.1007/s12072-023-10585-y.

## Introduction

Hepatocellular carcinoma (HCC) is the second leading cause of cancer-related deaths worldwide, and its incidence is expected to rise further [1]. Only 20% of patients with HCC are candidates for resection [2]. Over the past two decades, image-guided minimally invasive techniques have been widely used to treat hepatocellular carcinoma, and promising results have been obtained with one such technique, namely, radiofrequency ablation (RFA), with the survival rates comparable to those of hepatectomy [3]. Over the past decade, there has been increasing interest in RFA as a useful treatment modality for patients with HCCs that were considered unresectable due to poor liver function or complications [4, 5]. Improvements in surveillance programs have reduced the number of hepatocellular carcinomas detected in advanced stages, with a corresponding increase in the use of RFA [2].

While RFA is recognized as an effective local treatment for hepatocellular carcinoma, HCC was reported to be associated with a worse prognosis as tumor size increase, due to the high incidence of recurrence [6]. Risk stratification of HCC patients undergoing RFA can help clinicians identify patients at high risk for cancer recurrence/progression after RFA. These patients may require more aggressive treatments and closer monitoring to detect and manage cancer recurrence or progression. In addition, the economic and medical burden of HCC is significant, and more effective treatment methods are needed to prolong the survival and improve the quality of life of HCC patients. Therefore, effective tools are needed for prognostic evaluation of HCC patients undergoing RFA.

Machine learning (ML) is a branch of artificial intelligence (AI) drawing on the principles of computer science and mathematics, that is focused on developing and implementing computer algorithms through the development of probabilistic or statistical models based on existing data, to maximize the predictive accuracy in many fields [7]. Recently, tremendous success has been achieved in multiple fields, from natural language processing to computer vision through the use of transformer-based models [8–11]. Transformer models show improved performance over existing deep learning-based models, and are currently accorded state-of-the-art status for natural processing [10] and computer vision tasks [9, 11]. A novel transformer model that has been developed recently for survival analysis [12] has been demonstrated to show high performance on tabular data sets as compared with deep learning-based models, such as DeepSurv [13] and DeepHit [14]. Although the transformer model appears to be a promising technology for accurate evaluation of the prognosis in HCC patients treated by RFA, there are no reports yet of prediction of the prognosis using a transformer model in HCC patients undergoing RFA.

This study was aimed at developing and validating a transformer model for predicting the overall survival in patients with HCC treated by RFA, as compared with the performance of a deep learning-based model. Such a prediction model is expected to enable prediction of the therapeutic outcomes prior to treatment and allow physicians to design personalized therapeutic strategies for individual patients.

## Materials and methods

### Patients

Between February 1999 and December 2019, a total of 1855 treatment-naïve HCC patients underwent RFA as the initial treatment at the Department of Gastroenterology, The University of Tokyo Hospital. Information on patient age at the start of the initial treatment, sex, serum levels of albumin, total bilirubin (TB), aspartate aminotransferase (AST), alanine aminotransferase (ALT), serum creatinine, alpha-fetoprotein (AFP), and Lens culinaris agglutinin-reactive fraction of AFP (AFP-L3), platelet count, prothrombin time (PT), and results of serology for hepatitis B surface (HBs) antigen and hepatitis C virus (HCV) antibody status were available for all the 1855 patients. After excluding 49 patients in whom RFA had been performed with non-curative intent to reduce the tumor burden and 28 patients for whom information on the serum levels of des-gamma-carboxyprothrombin (DCP) was lacking due to warfarin administration, we enrolled 1778 patients in the present study. The inclusion criteria for RFA were as follows: platelet count not less than 50 × 10^3^/mm^3^, TB < 3 mg/dL, and prothrombin activity > 50%. Patients with macroscopic vascular invasion, refractory ascites, and/or extrahepatic metastasis were excluded from this study.

### Diagnosis of HCC

The diagnosis of HCC was accomplished through the use of dynamic computed tomography (CT) or magnetic resonance imaging (MRI), with hyper-attenuation during the arterial phase and washout during the late phase considered as conclusive criteria for the diagnosis of HCC [15]. In cases where the diagnosis of HCC could not be ascertained definitively by CT, an ultrasound-guided tumor biopsy was performed, with the pathological diagnosis made on the basis of the Edmondson–Steiner criteria [16].

### RFA procedure and follow-up

The RFA technique employed for the patients enrolled in this study is described elsewhere [17]. Briefly, RFA was carried out under real-time ultrasound guidance, using a single needle pass (Cool-tip^®^, Covidien, Boulder, CO, USA/Medtronic, Minneapolis, MN, USA, or VIVARF, STARmed^®^, Gyeonggi-do, Korea). Following treatment, patients were monitored for recurrence of HCC by dynamic CT or MRI at 4-month intervals; the serum AFP, DCP, and AFP-L3 levels were also recorded. Recurrence of HCC was diagnosed based on the same criteria as those applied for the diagnosis of HCC. Liver biochemistry tests were also conducted every 4 months to assess the liver function.

### Model development

We applied a transformer model for survival analysis (SurvTRACE) for predicting the overall survival in patients with HCC treated by RFA [12]. The transformer model is a type of machine learning architecture that uses self-attention to process input sequences of variable lengths and capture long-term dependencies. It has been accorded state-of-the-art status for various applications, including natural language processing, precluding the need to rely on traditional sequential processing methods [8–10]. To develop the model, 16 potential predictor variables of the patients were employed: age; sex; daily alcohol consumption; tumor number; maximum tumor size; serum albumin (Alb), TB, AST, ALT, AFP, DCP, and AFP-L3; serology test results for HCV antibody and HBs antigen; platelet count; and prothrombin time. We standardized the data to eliminate the influences of dimensional and value range differences among the variables. For comparison, we also developed a deep learning-based model using DeepSurv. DeepSurv is a multi-layer feedforward model that draws on biological neural networks for information processing. It estimates the impact of variables on the hazard rate parameterized by the network weight, thereby serving as a predictive tool in survival analysis [13].

### Statistical analysis

Continuous variables are presented as the median values with the first and third quartiles, while categorical variables are presented as numbers and frequencies (%). To evaluate the performance of the model, the 1778 patients enrolled in the study were randomly divided into three groups, as follows: (i) the training set (80%), which was used to build the model (1422 patients), (ii) the development set, which was used to determine when to stop the training process (178 patients), and (iii) the test set, which was used to evaluate the performance of each classifier (178 patients). The Harrell's *c*-index was determined to assess the discrimination performance of the models in both the training (internal validation) and test sets (external validation) [18]. All the prediction models were implemented and assessed in Python using SurvTRACE [12] and DeepSurv 0.2.1 [13]. The evaluative performance of each model was assessed by Kaplan–Meier curve analyses and log-rank tests across diverse risk groups using R3.6.3 (http://www.R-project.org) [19]. The significance level was set at < 0.05 for all tests.

## Results

### Patient characteristics

**Fig. 1:**
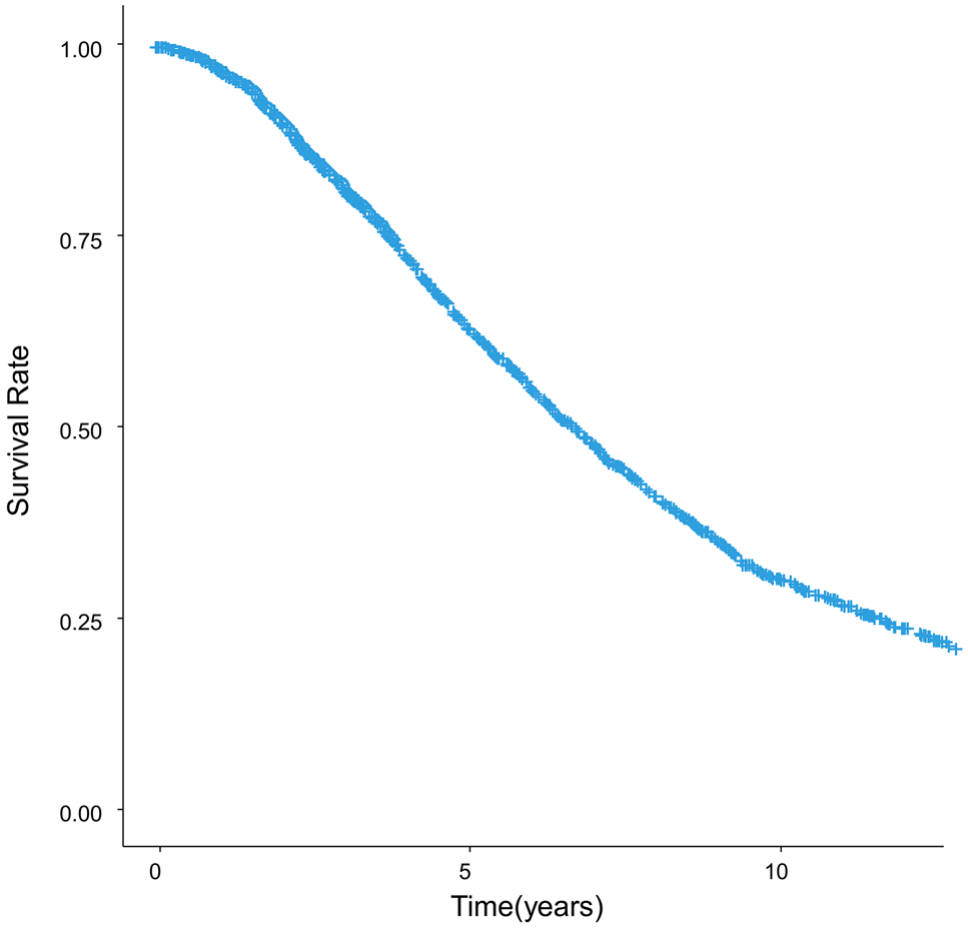
Overall survival in the 1778 primary hepatocellular carcinoma patients treated by radiofrequency ablation as the initial treatment

The study cohort consisted of 1778 treatment-naïve HCC patients who had undergone RFA as the primary treatment at a single institution. The patient characteristics are shown in Table 1; the median age was 70 years and approximately 60% were male. The median maximum tumor size and tumor number were 2.2 cm and 1, respectively. The patients with Child–Pugh class A accounted for 78.4% of all patients. Over a median follow-up of 50.7 months, the 5- and 10-year survival rates were calculated as 63.7% (95% confidence interval [CI] 61.4–66.1%) and 30.4% (95% CI 28.0–33.1%), respectively (Fig. 1). During the follow-up period, 1108 patients (62.3%) had died. The cause of death was HCC in 549 patients (49.5%), liver failure in 196 (17.7%), gastrointestinal bleeding in 41 (3.7%), complications related to procedure in 4 (0.4%), liver unrelated disease in 226 (20.4%), and undetermined in 92 (8.3%). The 1-, 3-, and 5-year local tumor recurrence with or without distant recurrence were 1.7%, 5.3%, and 6.5%, respectively (Supplementary Fig. 1).

**Table 1:** Baseline characteristics of the 1778 patients who underwent radiofrequency ablation treatment for primary hepatocellular carcinoma

Variable	Value (*n* = 1778)
Sex, *n* (%)	
Female	670 (37.7)
Male	1108 (62.3)
Age (years)	70 (64–76)
Body mass index	23.4 (21.3–36.7)
Viral infection, *n* (%)	
HBs antigen-positive	200 (11.2)
HCV antibody-positive	1219 (68.7)
Both positive	15 (0.8)
Both negative	344 (19.3)
Alcohol consumption, *n* (%)	
< 50 g/day	1346 (75.7)
50–80 g/day	171 (9.6)
> 80 g/day	261 (14.7)
Serum albumin (g/dl)	3.7 (3.4–4.0)
Serum creatinine (mg/dl)	0.7 (0.6–0.9)
Serum total bilirubin (mg/dl)	0.8 (0.6–1.1)
Prothrombin time (%)	86 (74–100)
Platelet count (× 10^3^/mm^3^)	110 (79–150)
Serum AST (IU/l)	50 (34.25–70.75)
Serum ALT (IU/l)	42 (27–66)
Tumor size (cm)	2.2 (1.7–2.9)
Tumor number	1 (1–2)
Serum AFP (ng/dl)	15 (6–56)
Serum DCP (mAU/ml)	22 (15–49)
Serum AFP-L3 (%)	0.5 (0.5–6.8)
Ascites, *n* (%)	
Absent	1563 (87.9)
Present	215 (12.1)
Encephalopathy, *n* (%)	
Absent	1741 (97.9)
Present	37 (2.1)

Continuous variables are represented as the median values with the first and third quartiles and categorical variables are represented as numbers and frequencies (%)

### Modeling process for developing the transformer model

For developing the transformer model, we applied the following hyperparameters: an epoch number of at most 100 with early stopping based on the development set performance, a batch size of 64, and a learning rate of 0.0001. The training process finished after 46 epochs, with a final validation loss of 1.16 (Supplementary Fig. 2).

### Predictive performance of the transformer model in comparison with that of the deep learning model

The discriminatory performance of the predictive models was assessed by determining the Harrell’s *c*-index. The index values for the test set (external validation) and training set (internal validation) were 0.69 and 0.71, respectively, for the transformer model, and 0.60 and 0.59, respectively, for the deep learning-based DeepSurv model (Table 2).

**Table 2:** Predictive performance (*c*-index) of the models

	Training set (internal validation)	Test set (external validation)
Transformer-based model	0.71	0.69
DeepSurv	0.59	0.60

### Prognostic stratification of the patients using the transformer model

**Fig. 2:**
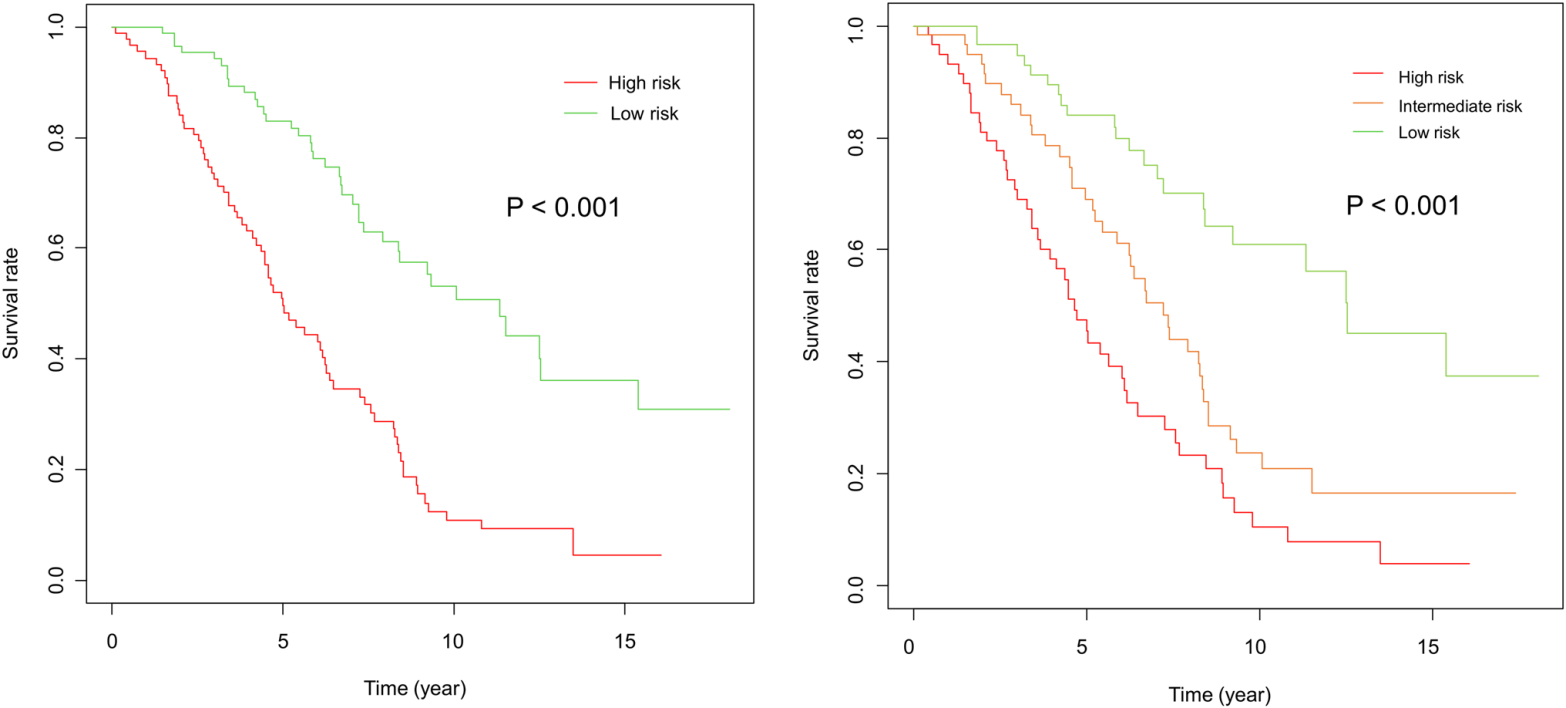
Kaplan–Meier curves for survival after initial RFA in the different risk groups—categorized into two (Fig. 1a) and three (Fig. 1b) risk groups using the transformer-based model

Figure 2 shows the Kaplan–Meier curves for survival time after the initial RFA categorized into two (Fig. 2a) and three (Fig. 2b) risk groups based on the survival estimation obtained using the transformer model. The transformer model showed a high discriminatory power for both the two and three risk categories (*p* < 0.001 in both risk categories).

### Personalized prediction using the transformer model

Finally, we applied the transformer model for predicting the overall survival in patients with HCC treated by RFA. Supplementary Fig. 2 shows the predicted survival in two patients: a case of a 76-year-old female patient who died about 6 years after the initial RFA treatment (Supplementary Fig. 3a) and a case of a 55-year-old female patient with confirmed survival of approximately 10 years after the initial RFA treatment (Supplementary Fig. 3b). The transformer model allowed output of individual survival predictions.

## Discussion

RFA plays an important role in the curative treatment of early-stage hepatocellular carcinoma because of its low invasiveness and high efficacy. The recent report from Italy showed a progressive patient aging, an incremental use of semi-annual surveillance which allowing for the detection of tiny lesions, and an increased use of radiofrequency ablation to the detriment of percutaneous ethanol injection from 2004 to 2018. Due to its minimally invasive nature and high efficacy in early-stage tumors, RFA plays an important role in the management of older patients and small hepatocellular carcinomas and may have influenced the ameliorative effect on cancer stage migration during this period[20].

Accurate prediction of the patient prognosis after RFA is a critical aspect of personalized medicine, as it is crucial for determining the most appropriate treatment plan for patients and improving the overall outcomes. For example, it can be hypothesized that patients characterized by the transformer model as having a negative prognosis are more likely to have highly malignant HCC. Such patients could potentially be optimal candidates for adjuvant or more intensive treatment. It may also aid in determining the frequency and intensity of follow-up monitoring/imaging. Several factors have been identified as prognostic indicators in HCC patients undergoing RFA, including the age, tumor size, tumor number, tumor marker, platelet count, and liver function [3, 21].

Despite the potential of the transformer model as a prognosis evaluation tool for HCC patients undergoing RFA, there are currently no reports available on its use as a prognostic prediction model in HCC patients treated by RFA; therefore, in this study, we developed a transformer-based machine learning model using potential risk factors to predict the overall survival in patients with HCC treated by RFA. To the best of our knowledge, this is the first study to report on the utility of a transformer model for predicting survival in patients with HCC. In the current study, we observed that the transformer-based model exhibited better predictive performance than DeepSurv, which had been identified previously as a robust model for predicting the survival or time-to-event [13].

The transformer model used in the current study is a neural network architecture that uses self-attention mechanisms and positional encoding to effectively model long-term dependencies in sequences of words or other tokens [8]. It has been accorded state-of-the-art status for a wide range of natural language processing tasks and is highly parallelizable, making it faster to train and able to handle larger datasets than earlier models [10]. For example, ChatGPT is a notable example of the advancement of transformer models for natural language processing and machine learning tasks [22], affirming the potential of the transformer model in various domains. The transformer model has also achieved state-of-the art status for multiple computer vision tasks [9, 11]. The ability of the transformer model to effectively model complex and long-term dependencies in data has made it a significant advancement in the field of machine learning and forecasting.

Personalization is one of the ultimate goals of modern medicine [23]. To achieve this, individualized risk prediction for each patient is critical. In the current study, we utilized the developed transformer model for predicting the prognosis in individual HCC patients undergoing RFA as the initial treatment. Notwithstanding the improved predictive performance of the transformer-based model developed in this study relative to that of the deep learning model, however, the prediction accuracy of the model is still not adequately satisfactory. The accurate prognosis prediction in HCC patients receiving RFA treatment was limited to fair to poor performance, with c-indices of 0.69 in the external validation site [24]. Lu et al. developed a prognostic nomogram for predicting the overall survival of HCC patients after RFA, and showed a *c*-index of 0.637 in the internal verification using the bootstrap method [25]. Accurate estimation of the survival in HCC patients undergoing RFA remains a challenge due to the significant variability of the clinical outcomes, which could also be influenced by non-hepatic comorbidities. Given that the predictive accuracy of machine learning models is influenced by the number of samples used for training [26], further research with larger sample sizes is necessary to enhance the accuracy of predictive models for clinical applications. Also, incorporating multiple heterogeneous forms of information, such as clinical data and imaging findings, through a multimodal representation model [27], could represent a valuable strategy to enhance the predictive performance.

Our study had several limitations. A primary limitation of this study was its single-center design, which restricts the generalizability of the findings—the predictive model developed in this study may not be applicable to other clinical settings. To confirm the general feasibility of the proposed transformer model, additional validation in a multi-center setting is necessary. Also, hospital volume was reported to be associated with the risk of mortality following RFA [28]. Therefore, the model can be expanded to include the hospital volume as a variable from the future multi-center study. Second, we have not publicly disseminated a web-based tool that facilitates access to the developed machine learning model. Consequently, the practical utilization of the model is currently precluded. What is significant, however, is that this is the first time that the transformer model has been demonstrated to work in the HCC field. Third, since the current study focused on RFA, biopsy was not performed for all cases, and therefore, the information regarding the differentiation of HCC is not available. Tumor location and shape of HCC were also important factors for treatment efficacy of RFA. The previous study showed an influence of tumor location on long-term outcomes following RFA. In this study, HCCs located in peri-portal-vein or peri-hepatic vein were associated with lower recurrence-free survival [29]. However, these factors were not included in the current study. Since we included the patients with multiple lesions, incorporating information on tumor localization and shape into machine learning models poses challenges due to the varying dimensions of these variables across different number of lesions. And finally, differences exist in the etiology of HCC between the current real-world setting and the study subject. The current study included patients who received radiofrequency treatment after 1999. Over the years, the prevalence of the underlying etiology of HCC has changed greatly. Although, recently, the proportion of HCC patients with non-viral etiologies has increased, reportedly accounting for about 30% of patients [30], only 20% of patients in the current study were non-viral patients.

In conclusion, we developed and validated a novel transformer model for personalized risk prediction of HCC recurrence/progression after RFA treatment. The current model has the potential to offer a personalized and precise schema for predicting the survival in HCC patients undergoing RFA treatment.

### Supplementary Information

Below is the link to the electronic supplementary material.

Supplementary file1 (TIF 394 KB)

Supplementary file2 (TIF 1685 KB)

Supplementary file3 (TIF 1313 KB)

Supplementary file4 (TIF 1245 KB)

Supplementary file5 (DOCX 15 KB)

## Data Availability

The data that support the findings of this study are available from the corresponding author, MS, upon reasonable request.

## Abbreviations

AFP: Alpha-fetoprotein
AFP-L3: Lens culinaris agglutinin-reactive fraction of AFP
ALT: Alanine aminotransferase
AST: Aspartate aminotransferase
CT: Computed tomography
DCP: Des-gamma-carboxyprothrombin
HBs: Hepatitis B surface
HCC: Hepatocellular carcinoma
HCV: Hepatitis C virus
ML: Machine learning
MRI: Magnetic resonance imaging
PT: Prothrombin time
RFA: Radiofrequency ablation
TB: Total bilirubin

## Declarations

### Guarantor of the article

Masaya Sato is the submission’s guarantor and takes responsibility for the integrity of the work as a whole, from inception to published article.
