# Supplementary figures and images for: Development of a transformer model for predicting the prognosis of patients with hepatocellular carcinoma after radiofrequency ablation
Source: Hepatol Int. 2023 Sep 9;18(1):131–7. doi: 10.1007/s12072-023-10585-y (PMC10857948; doi:10.1007/s12072-023-10585-y)

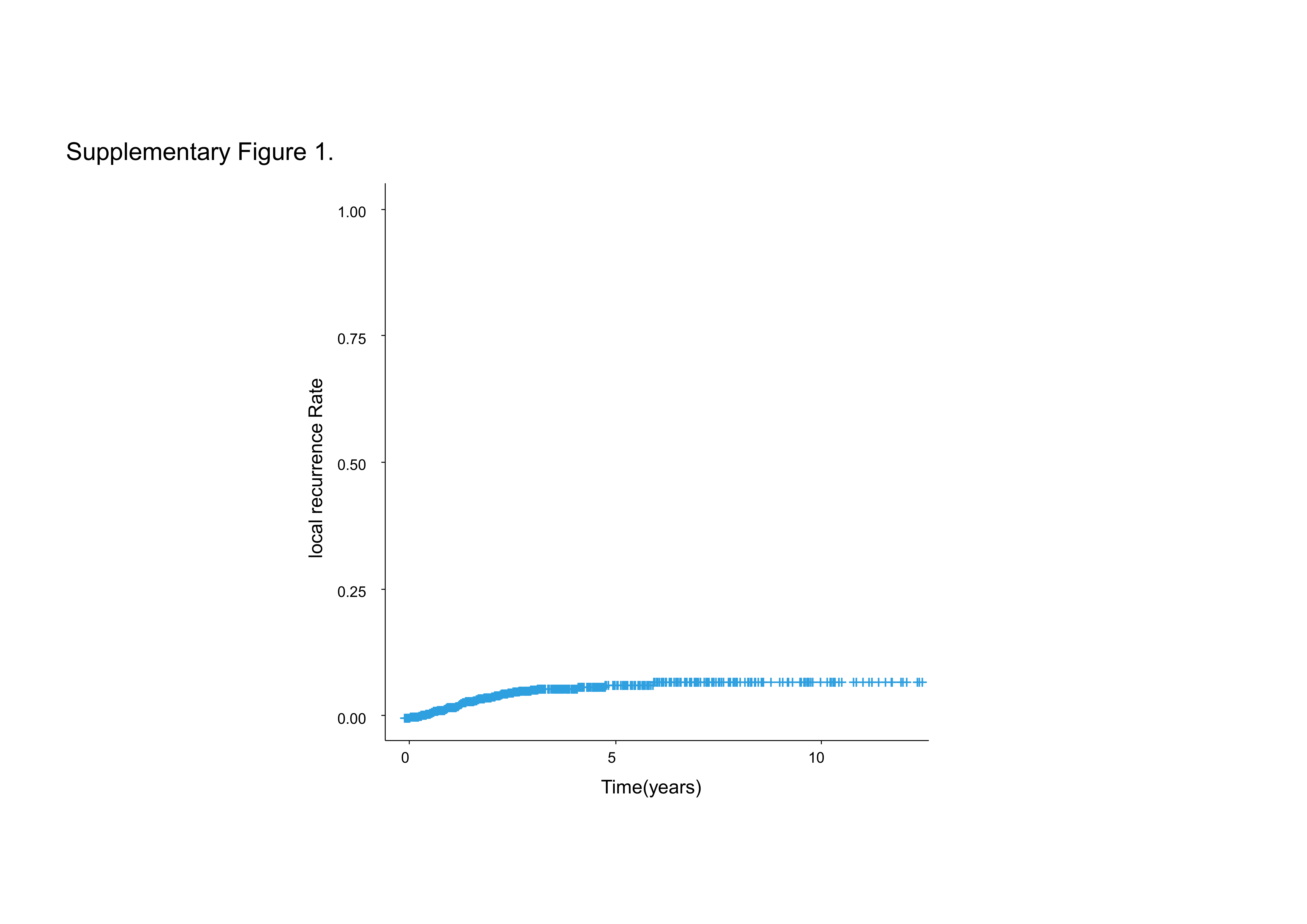

Supplement: Supplementary file 1 — Supplementary file1 (TIF 394 KB) [file 12072_2023_10585_MOESM1_ESM.tif]

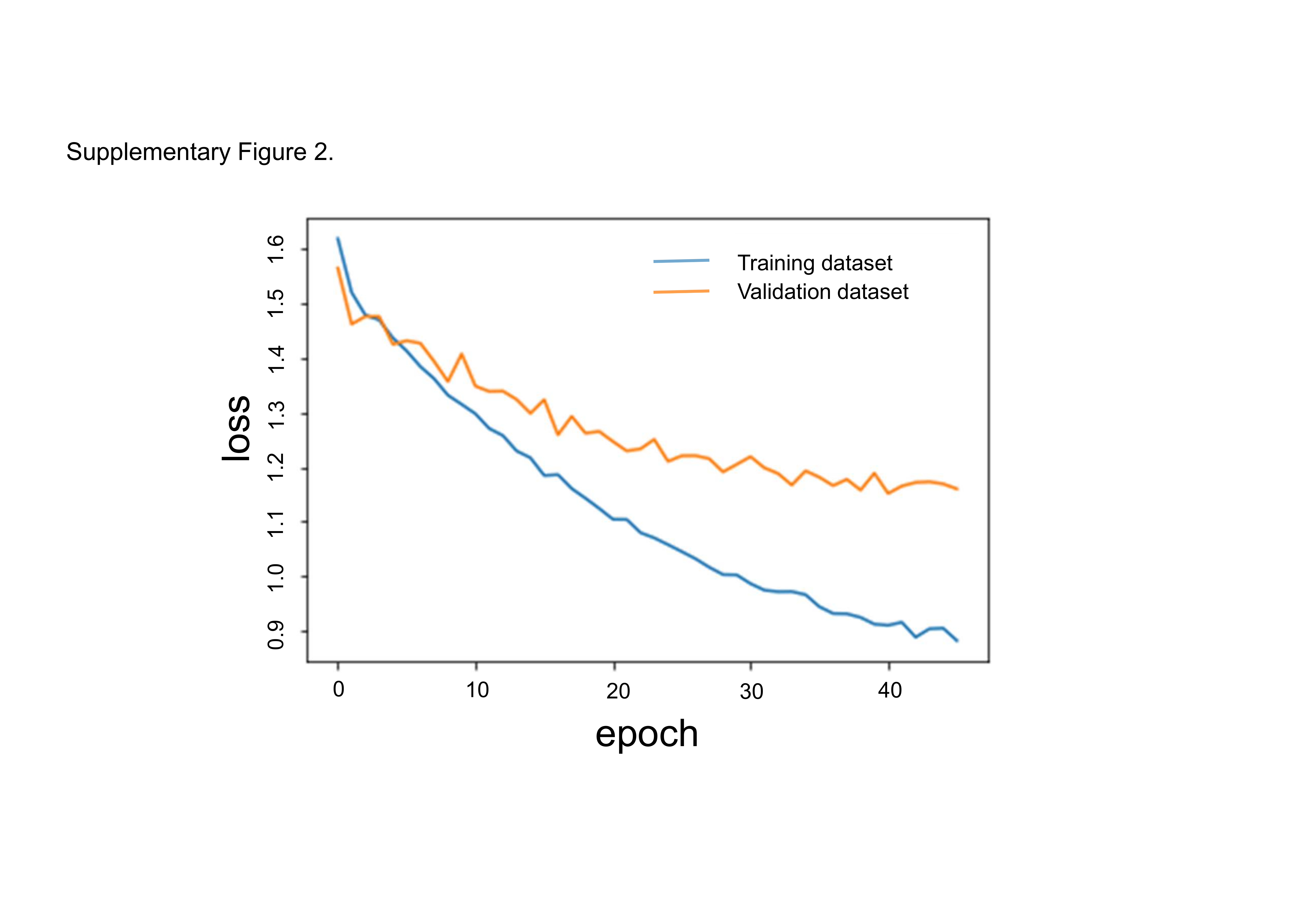

Supplement: Supplementary file 2 — Supplementary file2 (TIF 1685 KB) [file 12072_2023_10585_MOESM2_ESM.tif]

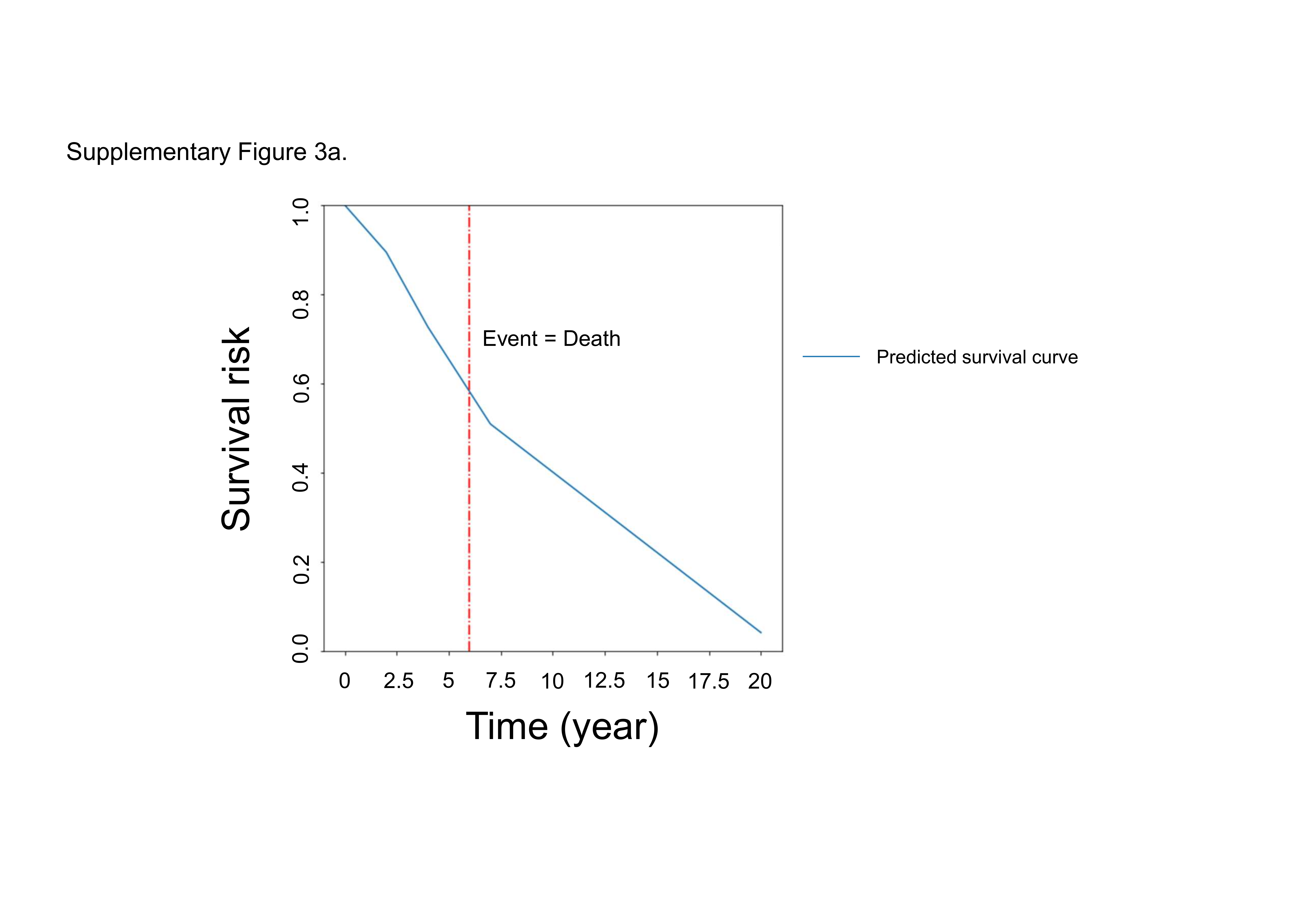

Supplement: Supplementary file 3 — Supplementary file3 (TIF 1313 KB) [file 12072_2023_10585_MOESM3_ESM.tif]

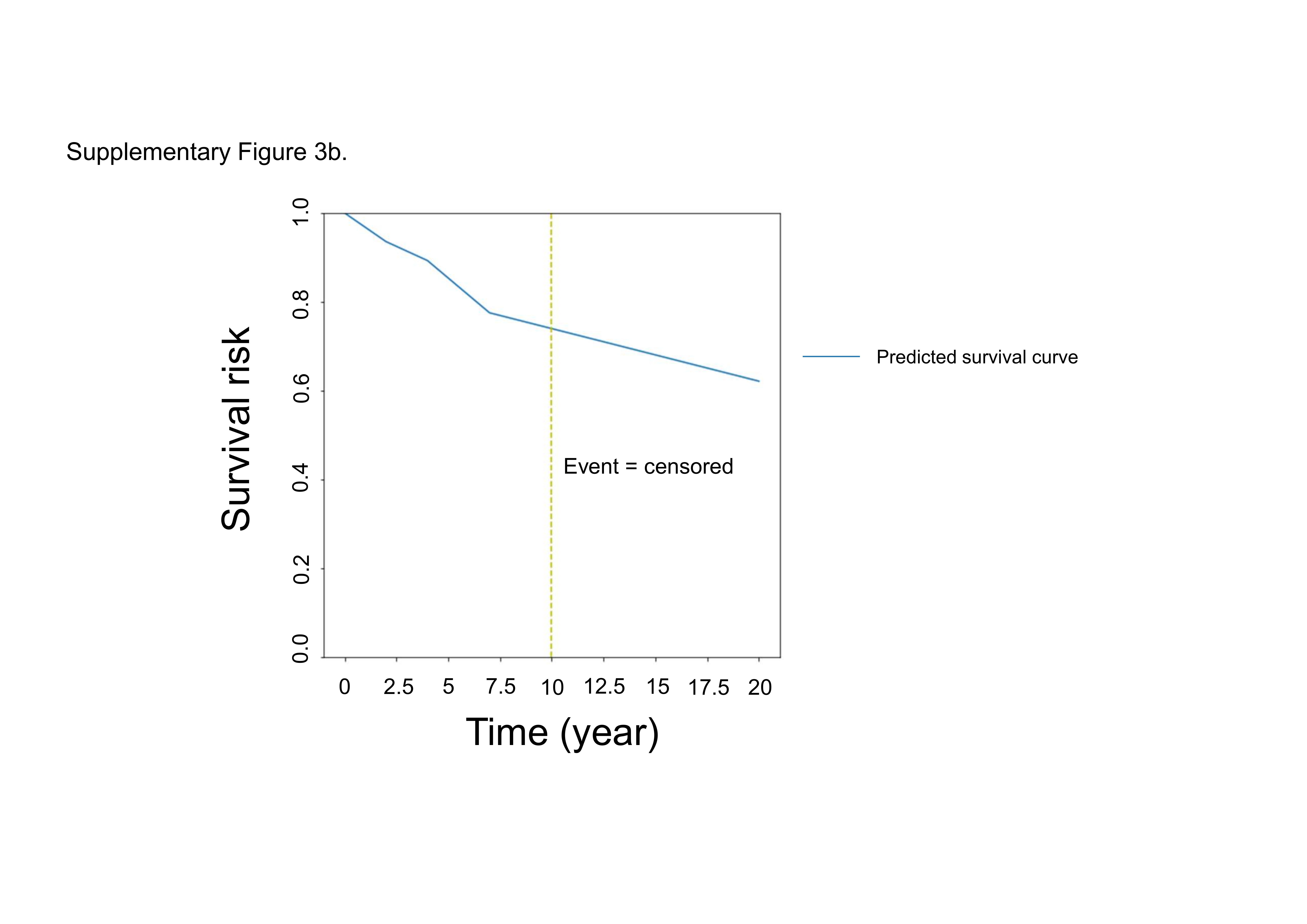

Supplement: Supplementary file 4 — Supplementary file4 (TIF 1245 KB) [file 12072_2023_10585_MOESM4_ESM.tif]
