# Supplementary material for: Development of a transformer model for predicting the prognosis of patients with hepatocellular carcinoma after radiofrequency ablation
Source: Hepatol Int. 2023 Sep 9;18(1):131–7. doi: 10.1007/s12072-023-10585-y (PMC10857948; doi:10.1007/s12072-023-10585-y)
Supplement: Supplementary file 5 — Supplementary file5 (DOCX 15 KB) [file 12072_2023_10585_MOESM5_ESM.docx]

# Supplementary Figure legends

Supplementary Figure 1. Local tumor recurrence in the 1,778 primary hepatocellular carcinoma patients treated by radiofrequency ablation as the initial treatment.

Supplementary Figure 2. Diagram of the training process for the transformer model, depicting loss function on the vertical axis and number of epochs on the horizontal axis.

Supplementary Figure 3. Personalized prediction of the prognosis in two patients - a case of a 76-year-old female patient who died about 6 years after the initial RFA treatment (Supplementary Figure 3a) and a case of a 55-year-old female patient with confirmed survival of approximately 10 years after the initial RFA treatment (Supplementary Figure 3b).
